# BRIDGING THE DIVIDE: REACHING THE POTENTIAL OF TECHNOLOGY TO ENHANCE OLDER ADULTS’ WELL-BEING AND QUALITY OF LIFE

**DOI:** 10.1093/geroni/igad104.0819

**Published:** 2023-12-21

**Authors:** Walter Boot

**Affiliations:** Florida State University, Tallahassee, Florida, United States

## Abstract

Existing and emerging technologies hold great promise with respect to improving the lives of older adults, in particular, by supporting and enhancing their independence, productivity, health, safety, social connectivity, and quality of life. Unfortunately, although differences in technology use and adoption between younger and older adults have declined over the past decades, barriers still exist that fully prevent the promise of technology from being realized to achieve these aims. This presentation will provide an overview of the current state of the intersection of aging and technology (nationally and internationally), with special attention to factors such as diversity, disability, and geographic region. Next, I will discuss approaches available to reduce the digital divide. These include changing the person (e.g., changing attitudes and proficiency) and changing the technology (human factors engineering). I will then discuss how a user-centered design process is crucial for the success of technology-based interventions. Without placing the user at the center of the design process, many novel technology solutions will likely fail to reach their potential. Finally, I will conclude with a discussion of if and when the age-related digital divide might close entirely by projecting changes in demographics, technology proficiency, technology proliferation, and technology change into the future.

